# Tumor suppressive microRNA-137 negatively regulates Musashi-1 and colorectal cancer progression

**DOI:** 10.18632/oncotarget.3726

**Published:** 2015-03-30

**Authors:** Amber R. Smith, Rebecca T. Marquez, Wei-Chung Tsao, Surajit Pathak, Alexandria Roy, Jie Ping, Bailey Wilkerson, Lan Lan, Wenjian Meng, Kristi L. Neufeld, Xiao-Feng Sun, Liang Xu

**Affiliations:** ^1^ Department of Molecular Biosciences, University of Kansas, Lawrence, KS, USA; ^2^ Department of Radiation Oncology, The Kansas University Medical Center, Kansas City, KS, USA; ^3^ Department of Oncology, and Department of Clinical and Experimental Medicine, Linköping University, Linköping, Sweden; ^4^ Department of Cancer Biology, The Kansas University Medical Center, Kansas City, KS, USA

**Keywords:** tumor-initiating cells, microRNAs, RNA-binding proteins, colon cancer, rectal cancer

## Abstract

Stem cell marker, Musashi-1 (MSI1) is over-expressed in many cancer types; however the molecular mechanisms involved in MSI1 over-expression are not well understood. We investigated the microRNA (miRNA) regulation of MSI1 and the implications this regulation plays in colorectal cancer. MicroRNA miR-137 was identified as a MSI1-targeting microRNA by immunoblotting and luciferase reporter assays. MSI1 protein was found to be highly expressed in 79% of primary rectal tumors (n=146), while miR-137 expression was decreased in 84% of the rectal tumor tissues (n=68) compared to paired normal mucosal samples. In addition to reduced MSI1 protein, exogenous expression of miR-137 inhibited cell growth, colony formation, and tumorsphere growth of colon cancer cells. Finally, in vivo studies demonstrated that induction of miR-137 can decrease growth of human colon cancer xenografts. Our results demonstrate that miR-137 acts as a tumor-suppressive miRNA in colorectal cancers and negatively regulates oncogenic MSI1.

## INTRODUCTION

Colorectal cancer (CRC) is the 3^rd^ most common cause of cancer-related deaths for men and women in the United States [1]. Worldwide, an estimated 1.4 million people were diagnosed with CRC in 2012 [2]. Although, screening methodologies have reduced the incidence of patients presenting with late stage disease at diagnosis, treating advanced stage colorectal cancer continues to challenge physicians. We are entering a new era of cancer research which depends on understanding the clinicopathological indexes paired with understanding the molecular footprint of cancer for improved tailored therapy, recently termed “precision medicine” [3, 4]. Recent evidence suggests that cancer originates from stem cell-like cells, termed tumor initiating cells (TICs) [5, 6]. It is believed that these stem-cell like cells are responsible for cancer initiation and also mediate metastasis and chemoresistance [6]. By gaining insight into the mechanisms of these rogue stem cell-like cells, targeted therapies can be designed against TICs.

Recently, a stem cell regulator, Musashi-1 (MSI1), was discovered to be highly expressed in colon primary tumors and metastatic lesions in the lymph nodes as compared to paired adjacent normal colon mucosal tissue [7]. Furthermore, this study identified MSI1 as a novel prognostic biomarker and therapeutic target for treating colon cancer [7]. Knocking down MSI1 in human colon cancer cell lines reduces growth, enhances apoptosis after radiation treatment [8] and reduces colon cancer proliferation, migration and invasion *in vitro* [7].

Normally expressed in stem cells, MSI1 is an RNA binding protein which can inhibit translation of target mRNAs, including that of adenomatous polyposis coli (*APC*), *NUMB* and cyclin-dependent kinase inhibitor/p21^WAF-1^ (*CDKN1A*) [9-11]. MSI1 represses translation by binding to the 3′ untranslated region (3′UTR) of target mRNA, therefore inhibiting formation of the 80S ribosome complex [12]. By down-regulating *NUMB*, *APC* and p21^WAF-1^, MSI1 positively regulates the Notch and Wnt signaling pathways and promotes cell cycle progression [9-11]. Though MSI1 has been identified as a therapeutic target, the molecular mechanisms responsible for overexpression of MSI1 in some colorectal cancers are not well understood. One possibility is a dysregulation of microRNAs (miRNAs) that negatively regulate *MSI1* mRNA.

miRNAs are short, 20-22 nucleotide, non-coding RNAs that regulate gene expression by binding to the 3′UTR of target mRNA thereby preventing protein translation or inducing mRNA destabilization [13]. miRNAs are predicted to target approximately 60% of all mRNAs, therefore, providing substantial regulatory power over many cellular processes [14]. The average 3′UTR length of miRNA target genes is approximately 1600 nucleotides, while non-miRNA target genes average 1000 nucleotides [15]. *MSI1* mRNA contains a long 3′UTR (~1800 nucleotides) consistent with possible post-transcriptional regulation by miRNAs. Recently, miRNAs negatively regulating *MSI1* mRNA were identified and found to be dysregulated in glioblastoma [16]. In that study, an initial list of putative *MSI1* targeting miRNAs was identified using the miRNA prediction program, TargetScan. Only the candidate miRNAs that had previously been reported to have implications in central nervous system tumors were examined for the ability to inhibit *MSI1*. This study provides substantial precedent that miRNAs regulate MSI1. We hypothesized that MSI1-regulating miRNAs are dysregulated in colorectal cancer, producing an over expression of MSI1. The objective of this project was to study the post-transcriptional regulation of MSI1 by miRNAs in colorectal cancer.

In this study, we show that miR-137 directly regulates MSI1. An inverse correlation between miR-137 and MSI1 is revealed in a panel of colon cancer cell lines. Additionally, we found that MSI1 protein expression is more abundant in rectal tumors compared to paired adjacent and distant normal mucosa. Alternatively, miR-137 levels are significantly decreased in rectal tumors compared to paired adjacent and distant normal mucosa. *In vitro* studies demonstrated that miR-137 over-expression decreases MSI1 expression, reduces cell growth, colony formation and tumorsphere growth. The restoration of miR-137 expression in xenograft tumor models also reduced tumor growth *in vivo*. Our work reveals a novel mechanism for MSI1 dysregulation in CRC and demonstrates miR-137 as a tumor suppressor miRNA.

## RESULTS

### Identification of MSI-targeting miRNAs

**Figure 1:**
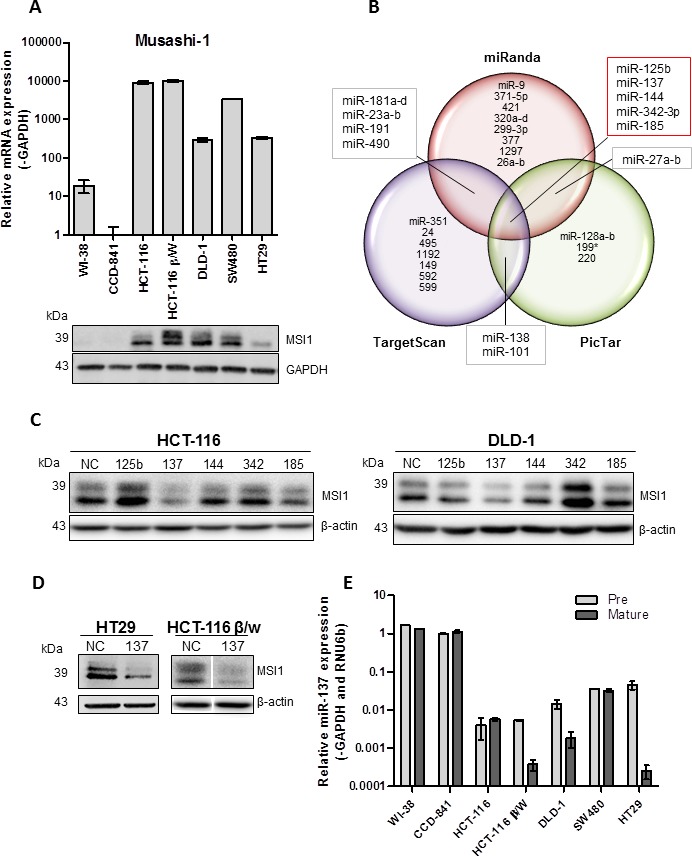
miRNA regulation of MSI1 (**A**) Expression of MSI1 mRNA and protein analyzed in a panel of colon cancer cell lines using quantitative real-time PCR and Western blotting. mRNA normalized to GAPDH and set relative to expression in normal colon epithelial cell line, CCD-841. (**B**) Venn diagram displaying highly conserved miRNAs predicted to bind to MSI1 3′UTR using miRanda, TargetScan and PicTar prediction software. (**C**) MSI1 protein expression analyzed in HCT-116 and DLD-1 colon cancer cell lines transfected with a panel of miRNA mimics as compared to cells transfected with a NC miRNA mimic. (**D**) MSI1 protein expression analyzed in HT29 and HCT-116 β/W colon cancer cells lines transfected with miR-137 mimic as compared to cells transfected with a NC miRNA mimic. (**E**) Precursor and mature miR-137 expression was analyzed in a panel of cell lines using qRT-PCR and Taqman PCR. Pre-miR-137 was normalized to GAPDH and mature miR-137 expression was normalized to RNU6b. Expression data was set relative to CCD-841.

MSI1 mRNA and protein is over-expressed in a panel of colon cancer cell lines compared to normal colon epithelial cell line, CCD-841 (Figure 1A). An apparent uncoupling of the mRNA and protein levels of MSI1 is revealed in this panel of cell lines, suggesting post-transcriptional regulation of MSI1. The molecular mechanism for MSI1 over-expression in colorectal cancer is not well understood. One possibility is a dysregulation of miRNA regulation. We utilized three miRNA targeting prediction programs (miRanda, TargetScan, PicTar) to identify highly conserved putative miRNA binding sites in *MSI1* 3′UTR. Using a variety of computational algorithms based on seed sequence position, pairing and conservation, these programs predict miRNA sites within target genes 3′UTR [17-19]. Among the three prediction programs, five overlapping miRNAs contained conserved, potential binding sites within *MSI1* 3′UTR; miR-125b, miR-137, miR-144, miR-185, and miR-342-3p (Figure 1B, Supplemental Table 1).

In order to determine which miRNAs negatively regulate MSI1 in colon cancer cell lines, miRNA mimics and a negative control (NC) mimic were transfected into high MSI1 expressing cell lines; HCT-116 and DLD-1. Exogenous expression of miR-137 reduced MSI1 protein levels compared to NC mimic in both HCT-116 and DLD-1 cell lines (Figure 1C). Interestingly, miR-125b and miR-342-3p mimics increased the expression of MSI1 in HCT-116 and DLD-1 respectively, suggesting an alternative mechanism of MSI1 regulation. Although this observation is beyond the scope of our current study, future studies focused on the miR-125b and miR-342-3p regulation of MSI1 may be of interest. Additional colon cancer cell lines HT29 and HCT-116 β/W were used to validate our findings, both of which displayed reduced MSI1 protein expression in cells transfected with miR-137 mimic (Figure 1D).

Since MSI1 is overexpressed in the panel of colon cancer cell lines, we hypothesized that miR-137 is down-regulated. We analyzed the expression of pre and mature-miR-137 in the same panel of colon cancer cell lines. In all five colon cancer cell lines examined, miR-137 expression was significantly decreased compared to the normal colon epithelial cell line, CCD-841 (Figure 1E). Normal human lung fibroblast cell line, WI-38, has similar miR-137 expression levels as the normal colon cell line, CCD-841. As expected, miR-137 and MSI1 expression are inversely correlated in cell lines (*P* = .04, Fisher Exact Test).

### miR-137 directly regulates MSI1

**Figure 2:**
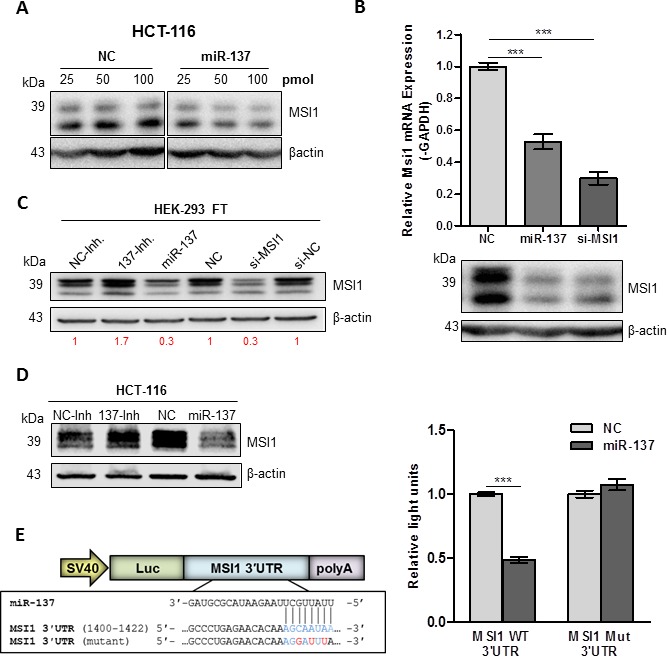
miR-137 negatively regulates MSI1 (**A**) MSI1 protein expression in HCT-116 cells transfected with increasing concentrations of miR-137 mimic as compared to cells transfected with NC mimic. (**B**) *Top,* MSI1 mRNA and protein expression analyzed in HCT-116 cells transfected with NC mimic, miR-137 mimic and positive control, MSI1-siRNA. mRNA expression analyzed using qRT-PCR, normalized to GAPDH and set relative to NC transfected cells. *Bottom,* MSI1 protein analyzed using Western blotting with β-actin as loading control. (**C**) MSI1 protein expression in HEK-293FT cells transfected with miR-137 antagomiR (137-Inh.), negative control antagomiR (NC-Inh.), miR-137 mimic (miR-137), negative control mimic (NC), MSI1 siRNA (si-MSI1) and a negative control siRNA (si-NC). Intensity of MSI1 protein was normalized to β-actin and set relative to control. Change in MSI1 protein shown in red. (**D**) MSI1 protein expression in HCT-116 transfected with NC-Inh, miR-137-Inh, NC-mimic and miR-137 mimic. (**E**) HCT-116 cells were transfected with wild-type (WT) or mutant (mut) pSGG-MSI1 3′UTR luciferase construct with miR-137 or NC mimic. Data are means ± SE; n = 3; *** *P* < 0.001.

Since miR-137 significantly decreased MSI1 protein expression in both HCT-116 and DLD-1 compared to the other mimics; we focused this study on understanding the miR-137-mediated regulation of MSI1. miR-137 reduced MSI1 protein expression in a dose-dependent manner (Figure 2A). Furthermore, miR-137 decreased *MSI1* mRNA levels more than cells transfected with NC mimic (*P* < .0001) and similarly as cells transfected with MSI1 siRNA (Figure 2B). Alternatively, inhibiting endogenous miR-137 in HEK-293FT and HCT-116 using antagomiRs increased MSI1 protein expression (Figure 2C and 2D).

Luciferase reporter assays were conducted to determine whether miR-137 inhibits MSI1 via the *MSI1* 3′UTR. HEK-293FT cells were co-transfected with *MSI1* 3′UTR luciferase reporter construct and miR-137 or NC mimic. As expected, miR-137 inhibited the luciferase expression of the *MSI1* WT 3′UTR construct (*P* < .0001), which was de-repressed by mutating the miR-137 seed sequence within the *MSI1* 3′UTR (Figure 2E). Our results confirm that miR-137 negatively regulates MSI1 via the *MSI1* 3′UTR.

### miR-137 down-regulates Wnt and Notch signaling

**Figure 3:**
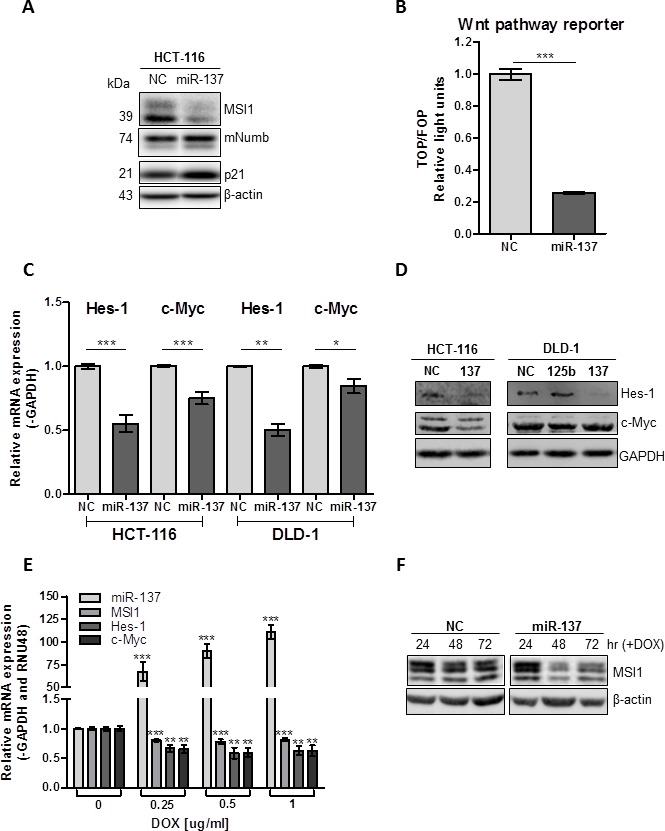
miR-137 negatively regulates Notch and Wnt signaling in colon cancer cell lines (**A**) MSI1, mNumb and p21 protein expression was analyzed in HCT-116 cells transfected with miR-137 and NC mimics. (**B**) HCT-116 cells were transfected with Top or Fop Flash constructs and miR-137 or NC miRNA mimic. Wnt signaling was stimulated with 20 mM LiCl for 16 h. Top Flash luciferase values were normalized to Fop Flash luciferase values. Data are means ± SE; n = 3; **P < 0.001. (**C**) mRNA expression of Hes-1 and c-Myc was analyzed in HCT-116 and DLD-1 cells transfected with miR-137 or NC mimic using qRT-PCR. Expression data was normalized to GAPDH and set relative to NC. Data are means ± SE; n = 3; *** *P* < 0.001, **P < 0.01, *P < 0.05. (**D**) Protein expression of c-Myc and Hes-1 in HCT-116 and DLD-1 cells transfected with NC mimic and miR-137 mimics. GAPDH is loading control. (**E**) Tet-on miR-137 HCT-116 cells were treated with increasing doses of DOX. Expression of mature miR-137 was analyzed using Taqman qRT-PCR, normalized to RNU48 and set relative to cells treated without DOX. MSI1, Hes1 and c-Myc mRNA was analyzed using qRT-PCR, normalized to GAPDH and set relative to cells treated without DOX. Data are means ± SE; n =3. (**F**) Tet-on miR-137 and NC HCT-116 cells were treated with 1 ug/ml DOX for 24, 48 and 72 hours. MSI1 protein was analyzed using Western blotting. β-actin used as loading control for Westerns.

If miR-137 successfully knocks down MSI1 levels, we would expect an increase in MSI1 target genes, p21 and mNumb. As expected, in miR-137-transfected HCT-116 cells, mNumb and p21 protein expression is increased compared to cells transfected with NC mimic (Figure 3A). MSI1 promotes cell growth by positively regulating Notch and Wnt signaling [20], therefore, we measured the change in both signaling pathways after miR-137 restoration. As measured by a β-catenin reporter (TOP/FOP) assay, miR-137 significantly reduced Wnt signaling in HCT-116 cells (*P* < .0001) (Figure 3B). We also measured Wnt and Notch signaling target genes, c-Myc and Hes-1, in miR-137- and NC-treated HCT-116 and DLD-1 cells. Both c-Myc and Hes-1 mRNA and protein expression were significantly reduced in miR-137-treated HCT-116 cells compared to cells treated with NC mimic (*P* = 0.0003 and *P* < .0001) (Figures 3C and 3D). Since the MSI1 target, APC is homozygous mutated in DLD-1 cell line and unable to interact with β-catenin, we expected to see a minimal change in Wnt signaling in this cell line. Notch signaling was reduced in DLD-1 cells, indicated by reduced Hes-1 mRNA (*P* = 0.002) and protein expression (Figures 3C and 3D). c-Myc mRNA (*P* = 0.0325) was slightly reduced in miR-137 treated DLD-1 cells; however there was no change in c-Myc protein expression in DLD-1 cells when treated with miR-137 (Figures 3C and 3D). Our data suggests that miR-137 reduces Wnt and Notch signaling pathways, in part by negatively regulating MSI1.

To study the effect of constitutively active miR-137, Tet-inducible miR-137 HCT-116 stable cells were produced using a pTRIPZ lentiviral expression vector. These stable clones are capable of inducing transcription of miR-137 when treated with doxycycline (DOX) to a physiological relevant level as compared to the expression of mature miR-137 in colon epithelial cell line, CCD-841 (Supplemental Figure S1). The addition of DOX results in decreased MSI1 expression and downstream Notch and Wnt signaling targets, Hes1 and c-Myc (Figure 3E). The decreased MSI1 expression is also observed at the protein level (Figure 3F). In summary, miR-137 restoration in colon cancer cell lines significantly reduced Wnt and Notch signaling; two important oncogenic signaling pathways regulated by MSI1 and involved in colorectal cancer progression.

### miR-137 acts as a tumor suppressor miRNA in colon cancer

**Figure 4:**
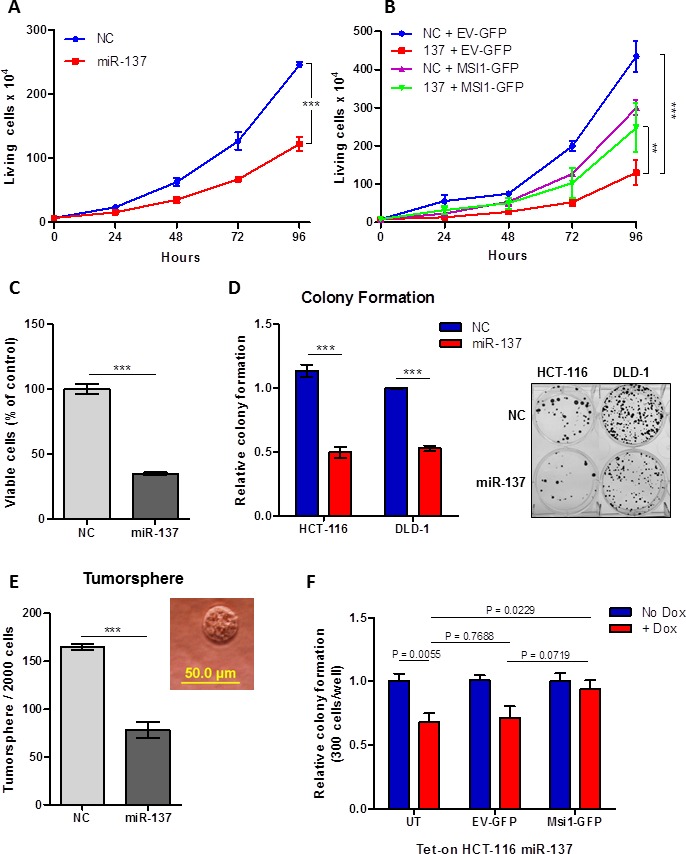
miR-137 inhibits colon cancer growth and clonogenic growth by inhibiting MSI1 (**A**) Cell growth curve in HCT-116 cells transfected with miR-137 and NC mimic. Cells were collected and counted every day for 4 days. Data are means ± SE; n = 3; *** *P* < 0.001. (**B**) Cell growth curve in HCT-116 cells co-transfected with miR-137/NC mimic and EV-GFP/MSI1-GFP expression vectors. Cells were collected and counted every day for 4 days. Data are means ± SE; n = 2; **P < 0.01, *** *P* < 0.001. (**C**) Cell viability was measured using a MTT colorimetric assay in HCT-116 cells transfected with miR-137 and NC mimic. (**D**) Colony formation assay in HCT-116 and DLD-1 cells transfected with miR-137 and NC mimic. Image of representative colonies are shown in right panel. (**E**) Tumorsphere assay in HCT-116 cells transfected with miR-137 and NC mimic. Data are means ± SE; n = 3; *** *P* < 0.001. (**F**) Colony formation on Tet-on miR-137 cells transfected with MSI1-GFP or EV-GFP constructs. Cells were plated in media with and without 1 μg/ml DOX. Data are means ± SE; n = 2; *P < 0.05, **P<0.01 and ***P < 0.001.

Based on our preliminary data, we hypothesized that miR-137 acts as a tumor suppressor miRNA by negatively regulating MSI1. To examine the effect of miR-137 restoration on colon cancer cells, *in vitro* cell growth and cell viability assays were utilized. miR-137 mimic transfected HCT-116 cells grew significantly less than cells treated with NC mimic (*P* < .0001) (Figure 4A). To examine whether the miR-137-induced cell growth inhibition was via MSI1 down-regulation, a phenotype rescue experiment was performed. miR-137 mediated inhibition of cell growth was partially restored when HCT-116 cells were co-transfected with a MSI1 cDNA expression vector that lacks the 3′UTR as compared to cells co-transfected with an empty vector (Figure 4B). This data suggests that miR-137 tumor suppressive function is mediated in part by negatively regulating MSI1. We also show that cell viability was significantly reduced upon miR-137 restoration as measured by a MTT cell viability assay (*P* < .0001) (Figure 4C).

Since MSI1 is a regulator of intestinal multipotent stem cells [21], we predicted that miR-137 reduces clonogenic cell growth in colon cancer. miR-137 significantly reduced clonal expansion of HCT-116 cells (*P* < .0001) and DLD-1 (*P* < .0001), as determined by a colony formation assay (Figure 4D). Similarly, tumorsphere growth was also significantly reduced by approximately 52% (*P* = .0006) upon miR-137 restoration in HCT-116 cells (Figure 4E). Tet-inducible miR-137 HCT-116 cells were transfected with either a MSI1 cDNA expression plasmid or control vector, in the presence or absence of DOX. In non-transfected cells, colonies grew significantly less when miR-137 expression was induced using DOX (*P* = .0055) (Figure 4F). However, when cells were transfected with MSI1 cDNA that lacks the 3′UTR, the colony formation capability was nearly completely restored (*P* = .0719) (Figure 4F).

In summary, miR-137 inhibits clonogenic growth, supporting its predicted role as a tumor suppressor miRNA that reduces colon cancer stem cell properties, in part by directly down-regulating MSI1.

### miR-137 reduces tumor growth *in vivo*

**Figure 5:**
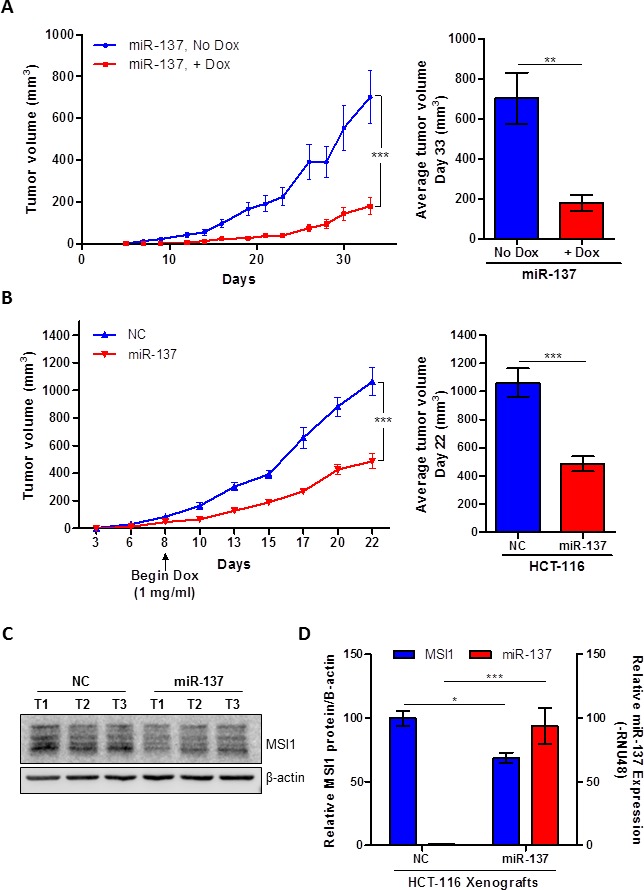
miR-137 inhibits human colon cancer xenografts growth (**A**) Tet-on miR-137 HCT-116 cells were injected into subcutaneously into mice. 10 mg/ml DOX was administered to the drinking water of one group of mice immediately following injections. Average tumor size was calculated at the end of the study and averaged for each group (on right). (**B**) Tet-on miR-137 and NC HCT-116 cells were injected into mice. After tumors grew to be approximately 50 mm^3^ in size, both groups of mice were given 10 mg/ml DOX in their drinking water. Average tumor size was calculated at the end of the study and averaged for each group (on right). (**C**) MSI1 protein expression was analyzed in three tumor samples from Tet-on miR-137 and NC HCT-116 xenograft samples. β-actin was used as loading control. (**D**) Relative mature miR-137 expression and relative MSI1 protein expression in xenografts tumor samples. Data are means ± SE; n = 3; *P < 0.05, **P < 0.01 and ***P < 0.001.

To study the effect of constitutively active miR-137 expression on tumor progression, HCT-116-miR-137 Tet-on stable clones were subcutaneously injected into flanking sides of athymic nude mice. Ten mice (mean weight = 18.68 g ± 0.563) were randomly separated into two groups. One group of mice received 10 mg/ml DOX in the drinking water immediately following injection, while the second group of mice did not receive DOX. The induction of miR-137 decreased tumor growth by approximately 75% (n = 8, *P* = .003, day 33) compared to the tumor growth in mice not fed DOX (n=10) (Figure 5A).

To control for DOX's effect on tumor growth, a separate animal experiment was performed using HCT-116 NC and miR-137 stable cells. Ten mice (mean weight = 22.1 g ± 0.458) were randomly separated into two groups. After cells were injected subcutaneously, both groups of mice were fed DOX in their drinking water once tumor volumes reached approximately 50 mm^3^. Similar to the previous animal study, the induction of miR-137 significantly inhibited colon cancer xenograft tumor growth by 55% (n=10, *P* < .0001, day 22), as compared to mice inoculated with inducible NC mimic (n=10) (Figure 5B).

Tumors were excised at the end of the study to analyze the expression of miR-137 and MSI1. The protein expression of MSI1 was decreased in the miR-137-treated tumors as compared to NC tumors (*P* = .0124) (Figure 5C and 5D). Additionally, miR-137 expression was significantly increased in the Tet-on miR-137 tumors compared to NC xenografts (*P* < .0001) (Figure 5D). In summary, induction of miR-137 significantly inhibited the human colon cancer xenograft tumor growth. Collectively the data supports our hypothesis that miR-137 acts as a tumor suppressor miRNA by down-regulating the oncogenic MSI1 that subsequently leads to tumor growth inhibition.

### Expression of MSI1 and miR-137 in patient tumor samples

**Figure 6:**
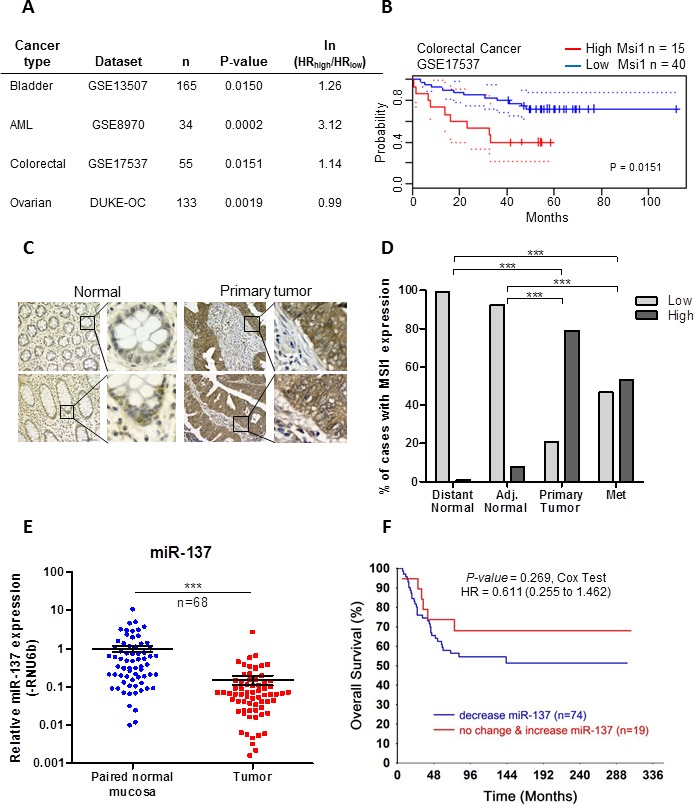
Musashi-1 is over expressed and miR-137 is decreased in rectal cancer tissue samples (**A**) Expression of MSI1 in a variety of cancer types is correlated with survival using PrognoScan database. (**B**) Kaplan-Meier curve associated with the colorectal dataset GSE17537. High MSI1 correlates with poor overall survival. Analyzed using PrognoScan database, Log-rank test, P = 0.0151. (**C**) Representative images of MSI1 immunohistochemistry in distant normal rectal mucosal tissues (left panel) and rectal primary tumor tissue (right panel). Bar = 5 μm. Magnification: 40x. (**D**) MSI1 intensity scores were categorized into low expressing (TMA scores of 0+1+2) and high expressing (TMA scores of 3). The percentage of low and high MSI1 expression was calculated for each tissue type. (**E**) Mature miR-137 expression was analyzed in 68 pairs of normal and primary rectal tumor tissues using Taqman PCR. miR-137 was normalized to RNU6b and set relative to matching normal. *** *P* < 0.001. (**F**) The overall survival of 93 patients was analyzed comparing decreased and increased expression of miR-137 using Kaplan-Meier survival analysis.

In order to understand the clinical relevance of our study, we used PrognoScan [22], a database used to correlate gene expression with patient prognosis, to determine the correlation between MSI1 expression with overall survival in patients with a variety of cancer types. Patients with high MSI1 expression was significantly correlated with an increased hazards risk for poor overall survival in patients with bladder cancer (n = 165, *P* = 0.0150), AML (n = 34, *P* = 0.0002), colorectal cancer (n = 55, *P* = 0.0151) and ovarian cancer (n = 133, *P* = 0.0019) (Figures 6A). In the colorectal dataset (GSE17537), high MSI1 significantly correlates with increased hazard risk, analyzed using a Kaplan-Meier curve and Cox proportional hazards regression test (Figure 6B).

We also examined the expression of MSI1 and miR-137 in available tissue samples from patients with rectal cancer. MSI1 protein expression was analyzed using immunohistochemistry in distant normal mucosa, adjacent normal mucosa, primary tumor and lymph node metastasis tissue samples collected from patients with rectal cancer (Figure 6C). The average score for MSI1 intensity was significantly higher in tumor samples collected from primary tumor (*P* < .0001) and metastatic lesions (*P* < .0001) (Supplemental Table 3) as compared to distant normal and adjacent normal mucosal tissue samples. For analysis, samples were categorized into high MSI1 expressing with intensity scores greater than two, while scores equal to or less than two were defined as low MSI1 expressing. MSI1 was highly expressed in 79% of primary tumor samples, and 53% of metastatic lesion samples. Inversely, MSI1 was highly expressed in 1% of distant normal samples and 8% of adjacent normal samples (Figure 6D and Supplemental Table 4).

The expression of miR-137 was measured in 68 paired normal mucosal and primary tumor tissue samples using Taqman qRT-PCR. miR-137 was significantly decreased in 84% of primary tumor tissue samples when compared to the paired adjacent normal tissues (n = 68, *P* < .0001) (Figure 6E and Supplemental Table 5). The expression of miR-137 did not statistically correlate with MSI1 expression in tissue samples (*P* = 0.54, Supplemental Table 6), although a clear trend is revealed in our patient data analysis and provides important precedence that analyzing more patients for the expression of MSI1 and miR-137 is needed in order to obtain statistical significance. Correlating miR-137 expression with patient survival demonstrated that patients with decreased miR-137 had an increased hazard of death (HR = 0.61) as compared to patients with no change or increased expression of miR-137, although this did not reach statistical significance (*P* = .269) (Figure 6F). In conclusion, our results show that miR-137 expression is decreased in colon cancer cell lines and rectal cancer tissues, and supports our overall hypothesis that loss of miR-137 promotes the overexpression of MSI1 in colorectal cancer.

## DISCUSSION

In the current study, we show that miR-137 can regulate MSI1 in colon cancer cell lines. We revealed an inverse correlation between miR-137 and MSI1 in a panel of colon cancer cell lines as compared to a normal colon epithelial cell line, and in primary rectal tumor tissues as compared to paired normal rectal mucosal tissue. miR-137 restoration in colon cancer cell lines, reduces MSI1 mRNA and protein levels, and inhibits cell growth, colony formation and tumorsphere growth. Furthermore, expressing miR-137 in HCT-116 significantly reduces xenografts tumor growth. In conclusion, our data suggest that miR-137 acts as a tumor suppressive miRNA and when down-regulated promotes tumorigenesis through the up regulation of MSI1.

Multiple studies have found an overexpression of MSI1 in a broad range of cancer types, including breast, lung and colon cancer [7, 8, 23, 24]. We are the first to report an overexpression of MSI1 specifically in rectal cancer in a large patient cohort. Although colon and rectal cancer are often grouped together under the category of colorectal cancer, their treatment strategies differ significantly. For colon cancer, typically surgical resection combined with chemotherapy is the primary treatment strategy [25]. For rectal cancer, radiation therapy combined with surgical resection is commonly used [25]. Previous studies have found that MSI1 knockdown sensitizes colon cancer cells to radiation therapy [8]. Collectively, this suggests that future therapeutic strategies targeting MSI1 may be relevant for both colon and rectal cancer patients and possibly in combination with conventional treatment strategies, such as radiation therapy. We hope to address these possibilities in future studies.

One aim of our study was to understand why MSI1 is overexpressed in colorectal cancer. As shown in the small intestine and colon tissue of adult and developing mice, MSI1 is predominately expressed in the base of crypts and less so towards the top of the transit amplifying region of the crypt, before reaching the villus location.[21] Stem cell genes are often tightly regulated by miRNAs upon differentiation [26, 27]. We propose that a lack of miRNA regulation during proliferation and subsequent differentiation of colon stem cell progeny causes MSI1 overexpression in colorectal cancer. The purpose of this study was to identify miRNAs capable of negatively regulating MSI1 in hopes of learning more about the potential cause of MSI1 overexpression in colorectal cancer. We successfully identified miR-137 as a negative regulator of MSI1 in colon cancer cells. Our findings are consistent with a previous study that discovered miR-137 as a negative regulator of MSI1 in a glioblastoma cell model [16].

Taken together, the data suggests that in normal cells, MSI1 is negatively regulated by miR-137, possibly during differentiation, and this level of regulation has been dismantled in colorectal cancer cells, thus leading to the MSI1 overexpression. Previous studies have shown that miR-137 expression increases upon differentiation of neural stem cells [28, 29] and mouse embryonic stem cells [30]. In cancer, miR-137 has been shown to be decreased in glioblastoma [29, 31], melanoma [32], gastric cancer [33], and colorectal cancer [34-36].

According to our results, loss of miR-137 appears to be at the transcriptional level since both pre and mature forms of miR-137 were decreased in colon cancer cell lines. Our data is consistent with previous studies that discovered loss of miR-137 occurs early in the progression of colon cancer due to hypermethylation of the promoter region [34], which prevents binding of the transcription factor, high-mobility group AT-hook (HMGA)1 [36]. This observation strongly supports our results and overall hypothesis that in colon cancer, miR-137 expression is silenced during differentiation due to hyper-methylation, resulting in MSI1 over-expression. However this specific mechanism needs to be studied further.

**Figure 7:**
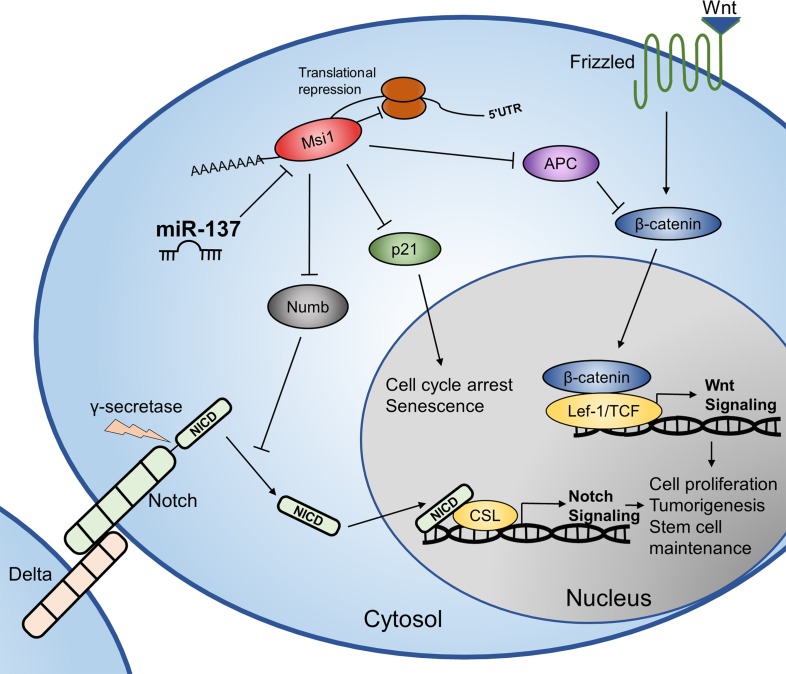
Working model RNA binding protein and stem cell regulator, MSI1, positively regulates the Wnt and Notch signaling pathways by binding to and inhibiting the translation of target mRNA; *APC*, p21^WAF-1^, and *NUMB*. In this study, we show that miR-137 acts as a tumor suppressive microRNA, in part by negatively regulating MSI1 and subsequently Wnt and Notch signaling pathways.

Overall, we describe an important tumor-suppressive mechanism of miR-137 through the negative regulation of MSI1 and Notch/Wnt signaling, outlined in our working model (Figure 7). miR-137 is a promising candidate for future miRNA-based molecular therapy for treating a variety of cancer types, including colorectal cancer. Our findings provide insight into the mechanism of dysregulation of colon cancer stem cells and eventually colorectal cancer initiation and progression. By understanding the molecular mechanisms of colorectal cancer biology, therapies may be developed to better combat this deadly disease.

## MATERIALS AND METHODS

### Patient samples and tissue microarray

Tissue microarray (TMA) slides were prepared from formalin-fixed paraffin embedded tissue blocks and contained tissue specimens from 146 primary rectal adenocarcinoma samples, 116 distant normal mucosa samples, 80 adjacent normal mucosa specimens, and 49 lymph node metastases. Tissue samples were collected from patients enrolled in a randomized clinical trial testing preoperative radiation therapy in the Southeast region of Sweden [37]. Distant normal mucosa was taken from proximal or distal margin (4-35 cm from the primary tumor) of the resected rectum and adjacent normal mucosa was taken from the mucosa tissue adjacent to the primary tumor. Both normal sections were histologically free from tumor. Additional details regarding the patient cohort and tumor characteristics are presented in Supplemental Table 2. Each patient was provided detailed information about the study aims and protocol, and gave their written informed consent prior to enrollment. The study was approved by the Institutional Review Board of the Linköping University, Sweden.

### PrognoScan analysis

The correlation between MSI1 expression and patient prognosis was evaluated using the PrognoScan database (http://www.abren.net/PrognoScan/) [22]. Using the minimum P-value approach, patients samples are grouped into high and low MSI1 expressing groups and correlated with survival using a Kaplan-Meier curve and log-rank test. Hazard risk is tested using a cox proportional hazards regression analysis.

### Immunohistochemistry of TMA

Immunohistochemistry was performed on TMA slides according to our previous publication [38]. Anti-Musashi-1 rabbit monoclonal antibody was used at a dilution of 1:50 (EMD Millipore, Billerica, MA, United States). A negative control experiment was performed using tissue samples from primary rectal cancer, incubated with PBS rather than anti-Musashi-1 primary antibody and probed with secondary anti-rabbit antibody to detect background signal produced by the secondary antibody (data not shown). The specificity of the anti-MSI1 antibody (Millipore, 04-1041) was tested in HEK-293FT cells treated with a MSI1-siRNA and NC-siRNA using Western blotting (data not shown). Specific details of the reagents and material used for this study are outlined in Supplemental Table 7.

Since MSI1 was homogeneously expressed in the epithelial cells among the tumor tissue sections, only MSI1 intensity was scored. Scoring was performed by two trained scientists independently without knowledge of clinicopathological information and reviewed by a pathologist. Each tissue sample had three replicate tissue cores included in the TMA. The highest TMA score for the three replicates was used as a representative score and the average intensity score between the two independent scorers was used as a final representative TMA score for each tissue sample. Scoring was based on MSI1 intensity: scores 0 displayed no visible MSI1 staining (negative), scores 1, 2 and 3 were weak, moderate and strong MSI1 staining.

### Taqman microRNA analysis in patient tissue samples

MicroRNA was isolated from paraffin-embedded tissue sections from rectal cancer patients using RecoverAll™ Total Nucleic Acid Isolation Kit for FFPE (Life Technologies, Stockholm, Sweden). The microRNA quality was assessed by studying amplification efficiencies of microRNAs after serial dilutions of cDNA and measuring CT values of microRNAs using real time PCR. miR-137 expression was determined using Taqman microRNA assay and normalized to control small RNA, RNU6b. The expression of miR-137 in the tumor samples was normalized to RNU6b and set relative to the expression of miR-137 in normal mucosal samples.

### Cell culture and reagents

The following cell lines were purchased from American Type Culture Collection (ATCC, Manassas, VA, United States); HCT-116, DLD-1, SW480, HT29, 293 FT, 293 WT, CCD-841 CoN and passaged according to ATCC protocol. HCT-116 β/W cell line was a generous gift from Bert Vogelstein (The Johns Hopkins University School of Medicine) [39]. Cells were cultured in Dulbecco's modified Eagle medium (Sigma-Aldrich, St. Louis, MO, United States), supplemented with 10% fetal bovine serum (GE Healthcare HyClone, Logan, Utah, United States), and 1% Penicillin-Streptomycin antibiotic (Life Technologies, Grand Island, NY, United States).

### *In vitro* studies

miRNA mimics, siRNAs, NC mimics, NC siRNA, miR-137 antagomiR and antagomiR-NC were purchased from Dharmacon (GE Dharmacon, Lafayette, CO, United States). Transfections were carried out as previously described [40]. Briefly, cells plated in a 6-well plate were transfected with 100 pmol (50nM) miRNA mimics, siRNAs or antagomiRs using Lipofectamine 2000 (Invitrogen, Life Technologies). For cell growth assays, HCT-116 cells were transfected with miR-137 mimic and negative control (NC) mimic and re-seeded 24 hours later in a 24-well plate. Cells were collected every day for 4 days and living cells were counted using trypan blue staining and a hemocytometer as previously described [41]. For the cell growth rescue experiment, cells were co-transfected with miR-137 and NC mimics (50nM) and the MSI1-GFP/EV-GFP expressing constructs (1 μg) for 24 hours and re-seeded in a 24-well plate. Cells were collected and counted as described above every day, for 4 days. Cell viability of cells was determined using a MTT colorimetric assay. Briefly, cells were transfected with miR-137 and NC mimics, 24 hours later cells were re-seeded into a 96-well plate in triplicate. After 4-6 days (or until ~90% confluency of NC group), cell medium was replaced with WST-8 (Sigma) dye for 1-5 hours. The absorbance was quantified using a microplate reader (BioTek, Winooski, VT, United States) at 450 nm. The absorbance of miR-137-treated cells was set relative to cells treated with NC mimic.

### Western blot analysis

After 48-72 hours of transfection with mimics, antagomiRs, or siRNAs, cells were collected for Western blotting as previously described [42]. Antibodies used for our studies are detailed in Supplementary Table 7.

### Luciferase reporter assays

The pSGG-MSI1 3′UTR luciferase construct was a gift from Luiz O.F. Penalva and previously described [43]. Cells were transfected with 200 ng of pSGG-MSI1 3′UTR luciferase construct and 20 nM miR-137 mimic or NC mimic using Lipofectamine 2000. Three nucleotides within the miR-137 seed sequence in the pSGG-MSI1-3′UTR construct were mutated using the QuikChange Site-Directed Mutagenesis Kit (Agilent, Santa Clara, CA, United States). After 48 hours, cells were harvested and assayed using the Dual-Luciferase Reporter Assay System (Promega, Madison, WI, United States). Wnt signaling was measured using the TOP/FOP Flash reporter constructs (Millipore). Cells were transfected with 200 ng of TOP or FOP Flash constructs and 20 nM miR-137 mimic or NC mimic using Lipofectamine 2000. Transfected cells were stimulated with 20 mM LiCl for 16 hours prior to harvest at 48 hours post-transfection. A renilla luciferase construct was used as a normalizing control for all luciferase assays. Experiments were conducted in triplicate.

### Quantitative real-time PCR

Quantitative real-time PCR was performed using gene specific primers (Supplemental Table 8), SYBR^®^ Select master mix (Applied Biosystems, Life Technologies) and normalized to GAPDH. The expression of mature miR-137 was quantified using microRNA TaqMan® Assays (Life Technologies) and normalized to housekeeping small RNAs, RNU6b or RNU48 (Supplemental Table 7). Expression of genes were normalized using the equation 2^−ΔCt^ = 2^−(Ct (housekeeping gene) – Ct (gene of interest))^ [44].

### Colony formation and tumorsphere assay

Colony formation assays were performed using HCT-116 cells transfected with 100 pmol mimics (miR-137 and NC) in a 6-well plate for 24 hours and then re-seeded (300 cells/well) in a 6-well plate in triplicate according to our previous publication [40]. For the tumorsphere assay, HCT-116 cells were transfected as described above and re-seeded into 24-well ultra-low attachment plates in triplicate at 2000 cells per well according to our previous publication [45]. The number of colonies and tumorspheres grown in miR-137 treated cells was set relative to the number of tumorspheres grown in NC treated cells. A rescue experiment was performed using a colony formation assay and a pCMV6-MSI1-GFP construct purchased from Origene (Rockville, MD, United States). A pCMV6-EV-GFP construct was produced by removing the MSI1 coding sequence from Sgf1/Xho1 sites and replacing it with a 24 base pair linker; 5′-TCACAACCTCCTAGAAAGAGTAGA-3′.

### Production of Tet-on HCT-116 stable cell lines

The mature miR-137 or NC mimic sequence was cloned into the Tet-inducible pTRIPZ expression vector (Dharmacon) according to manufacturer's instructions. Stable cell lines were generated by lentiviral transduction of HCT-116 cells and selected with 1.2 μg/ml puromycin for 1 week. Cells were treated with 0.25, 0.5 or 1 μg/ml of doxycycline (DOX) to induce the transcription of miR-137 in HCT-116.

### Animal studies

Animal experiments were done according to the Institutional Animal Care and Use Committee (IACUC) protocol approved by the University of Kansas Guidelines for Use and Care of Animals. HCT-116 stable clones with inducible miR-137 and NC miRNA were injected (0.5 × 10^6^) into sub-cutaneous regions 5-6 week old, female athymic nude mice (Hsd:Athymic Nude-Foxn1^nu^, Harlan). Mice were separated into control and experimental groups randomly (N=5/group) for each animal study. In the tumor progression experiment, after the tumors reached approximately 50 mm^3^ in size, mice were fed 1 mg/ml Doxycycline Hyclate (Sigma) in the drinking water to induce the expression of miR-137 or NC miRNA. In the tumor initiation study, DOX (1 mg/ml) was administered immediately after cell injections. Tumor growth was measured using calipers, 2-3 times per week. To avoid biased interpretations, scientists measuring tumor volume were unaware of treatment groups. Tumor volume was calculated using the following equation: (length x width^2^)/2.

### Statistical analysis

Chi-square test was used to examine the significance of the differences in MSI1 expression in distant or adjacent normal mucosa, primary cancer and lymph node metastasis, as well as the correlation of MSI1 expression and miR-137 expression with clinicopathological variables. miR-137 expression with overall survival was tested using Log-Rank and Cox proportional hazards regression analysis. Survival curves were computed according to Kaplan-Meier estimates. Two-way ANOVA test was utilized to study the significance of the cell growth curve and xenograft tumor growth studies. All tests were two sided and a P-value (*P*) of < .05 was considered statistically significant. Data represent average results from at least three independent experiments and shown as the mean ± SE. *P < .05; **P < .01; and ***P < .001.

## SUPPLEMENTARY MATERIAL, FIGURE AND TABLES


