# Supplementary material for: Tumor suppressive microRNA-137 negatively regulates Musashi-1 and colorectal cancer progression
Source: Oncotarget. 2015 Mar 30;6(14):12558–73. doi: 10.18632/oncotarget.3726 (PMC4494958; doi:10.18632/oncotarget.3726)
Supplement: Supplementary file 1 [file oncotarget-06-12558-s001.pdf]

Tumor suppressive microRNA-137 negatively regulates Musashi-1 and colorectal cancer progression

Supplementary Material

Supplemental Table 1: Alignment of Predicted miRNAs in MSI1 3′UTR

<table>
<tr><th>miRNA</th><th>Sequence Alignment</th><th></th></tr>
<tr><td>miR-125b
(1090-1097)</td><td>5′ ...GAAAGCAAAGGGCGUCUCAGGGA...
|||||||
3′ AGUGUUCAAUCCCAGAGUCCCU</td><td>MSI1 3′UTR

miR-125b</td></tr>
<tr><td>miR-137
(1415-1422)</td><td>5′ ...GCCCUGAGAACACAAAGCAAUAA...
|||||||
3′ GAUGCGCAUAAGAAUUCGUUAUU</td><td>MSI1 3′UTR

miR-137</td></tr>
<tr><td>miR-144
(779-785)</td><td>5′ ...CCCCUCAGACACCGUUACUGUAA...
||||||
3′ UCAUGUAGUAGAUAUGACAU</td><td>MSI1 3′UTR

miR-144</td></tr>
<tr><td>miR-342-3p
(1378-1384)</td><td>5′ ...GAGAGUUAAUUGUCUGUGUGAGG...
|||||||
3′ UGCCCACGCUAAAGACACACUCU</td><td>MSI1 3′UTR

miR-342-3p</td></tr>
<tr><td>miR-185
(943-949)</td><td>5′ ...CUGGACAGGAAUUAACUCUCCAA...
||||||
3′ AGUCCUUGACGGAAAGAGAGGU</td><td>MSI1 3′UTR

miR-185</td></tr>
<tr><td>miR-185
(1722-1729)</td><td>5′ ...UCCUCCGGUGUCUCUUCUCUCCA...
|||||||
3′ AGUCCUUGACGGAAAGAGAGGU</td><td>MSI1 3′UTR

miR-185</td></tr>
</table>

Supplemental Table 2. Patient and Tumor Characteristics

Characteristics	*MSI1* TMA (%)	P-value [a]	miR-137 (%)	P-value [a]
Sex				
Male	87 (60)	0.20	60 (65)	0.36
Female	59 (40)		33 (35)	
Age				
≤ 70	89 (61)	0.24	57 (61)	0. 59
> 70	57 (39)		36 (39)	
TNM				
I	42 (29)	0.57	30 (32)	0.39
II	41 (28)		24 (26)	
III	54 (37)		32 (34)	
IV	9 (6)		7 (8)	
Growth Pattern				
Expansive	87 (69)	0.22	53 (65)	0.57
Infiltrative	40 (31)		29 (35)	
Differentiation				
Good	3 (2)	0.39	2 (2)	0.72
Med	113 (77)		70 (75)	
Poor (poor + mu + sig)	30 (21)		21 (23)	
Local-Recurrence				
No	124 (85)	0.30	76 (82)	0.66
Yes	22 (15)		17 (18)	
Distant-Recurrence				
No	88 (60)	0.44	53 (57)	0.44
Yes	58 (40)		40 (43)	

[a] Determined using Chi-Square test.

Supplemental Table 3. Musashi-1 Staining Intensity in Distant Normal, Adjacent Normal, Biopsy, Primary Tumor and Metastatic Rectal Cancer Tissue Samples

	MSI1 Staining Intensity[a]				
**Tissue Type**	**-**	**+**	**++**	**+++**	**Total N**
Normal	22 (18.6%)	65 (55%)	30 (25.4%)	1 (0.8%)	118
Adjacent Normal	8 (10%)	32 (40%)	34 (42.5%)	6 (7.5%)	80
Primary	0	3 (2%)	28 (19%)	115 (78.7%)	146
Met	0	9 (28.6%)	14 (28.6%)	26 (53%)	49

[a] MSI1 TMA scores of 0,1,2,3 are defined as -, +, ++, +++.

Supplemental Table 4. Musashi-1 Expression in Distant Normal, Adjacent Normal, Biopsy, Primary Tumor and Metastatic Rectal Cancer Tissue Samples (low vs. high)

	MSI1 Expression[a]		
**Tissue Type**	**Low**	**High**	**Total N**
Normal	117 (99%)	1 (1%)	118
Adjacent Normal	74 (92.5%)	6 (7.5%)	80
Primary	31 (21%)	115 (79%)	146
Met	23 (47%)	26 (53%)	49

[a] TMA scores of 0+1+2 are defined as low expression and TMA scores of 3 are defined as high expression

Supplemental Table 5. miR-137 Expression in Normal Rectal Tissues and Primary Tumor Rectal Tissue Samples

	miR-137 Expression[a]			
**Tissue Type**	**No Change**	**Decreased**	**Increased**	**Total N**
Matched Primary[a]	2 (3%)	57 (84%)	9 (13%)	68
Unmatched Primary[b]	0	22 (73%)	8 (27%)	30
Total Primary[c]	2(2%)	79 (81%)	17 (17%)	**98**

[a] miR-137 expression in primary tumor tissue sample was normalized to RNU6b and set relative to the expression of miR-137 in matching normal tissue sample.

[b] miR-137 expression in primary tumor tissue sample was normalized to RNU6b and set relative to the average expression of miR-137 in unmatched normal tissue samples.

[c] Combined change in miR-137 expression in paired and un-paired primary tumor tissues as compared normal mucosal tissues.

Supplemental Table 6. Correlation of miR-137 and Musashi-1 Expression in Primary Rectal Tumor Tissue Samples

	miR-137 Expression[a]				
**MSI1**	**No Change**	**Decreased**	**Increased**	**Total N**	**P-value**[d]
Low[b]	1 (6%)	13 (76%)	3 (18%)	17	0.54
High[c]	1 (1%)	57 (81%)	12 (17%)	70	

[a] miR-137 expression in primary tumor tissue sample was normalized to RNU6b and set relative to the expression of miR-137 in normal tissue sample.

[b] MSI1 TMA scores of 0+1+2.

[c] MSI1 TMA scores of 3.

[d] Determined using Chi-Square test, χ^2, df = 1.224, 2

Supplemental Table 7. Antibodies and Reagents

Product	Catalog #	Supplier	Comments
**Antibodies**			
Rabbit anti-Musashi1	5663	Cell Sign.	1:500
Mouse anti-GAPDH	sc-51905	Santa Cruz	1:500
Rabbit anti-mNumb	2756	Cell Sign.	1:500
Rabbit anti-p21	sc-397	Santa Cruz	1:500
Rabbit anti-Hes1	Ab71559	Abcam	1:500
Rabbit c-Myc	5605	Cell Sign.	1:1000
Mouse anti-β-actin	A5316	Sigma	1:5000
Mouse anti-GAPDH	Sc-51095	Santa Cruz	1:500
Goat anti-mouse, peroxidase conjugated	31430	Fisher	1:8000
Goat anti-rabbit, peroxidase conjugated	31460	Fisher	1:8000
Goat anti-mouse, IRDye® 680RD	926-68070	LI-COR	1:15000
Goat anti-rabbit, IRDye® 800CW	926-32211	LI-COR	1:15000
Rabbit anti-Musashi-1 (for IHC)	04-1041	Millipore	1:50
Anti-Rabbit, peroxidase conjugated (for IHC)	K4010	Dako	
**Reagents**			
Hematoxylin	S3302	Dako	
EnVision+ System-HRP (for IHC)	K4010	Dako	
Doxycycline Hyclate	D9891	Sigma	
SYBR® Select Master Mix	4472908	AB	
Lipofectamine 2000	11668019	LT	
TRIzol	15596	LT	
**Plasmids**			
pTRIPZ		Dharmacon	
pCMV6-MSI1-GFP	RG215992	Origene	
NC-GFP		Origene	Modified
Renilla control vector	E2241	Promega	
TOP/FOP vectors	21-170/21-169	Millipore	
**Mimics/siRNAs/AntagomiRs**			
hsa-miR-125b miRIDIAN mimic	C-300595-03	Dharmacon	100 nM
hsa-miR-137 miRIDIAN mimic	C-300604-07	Dharmacon	100 nM
hsa-miR-144 miRIDIAN mimic	C-300612-05	Dharmacon	100 nM
hsa-miR-342-3p miRIDIAN mimic	C-300696-05	Dharmacon	100 nM
hsa-miR-185 miRIDIAN mimic	C-300636-07	Dharmacon	100 nM
NegCtl. #1, miRIDIAN mimic	CN-001000-01	Dharmacon	100 nM
hsa-miR-137, miRIDIAN Hairpin Inhibitor	IH-300604-08-005	Dharmacon	100 nM
NegCtl. #1, miRIDIAN Hairpin Inhibitor	IN-001005-01	Dharmacon	100 nM
Human Msi1 siRNA	J-011338-08	Dharmacon	100 nM
Non-targeting-siRNA #1	D-001810-01	Dharmacon	100 nM
**Primers**			
Taqman hsa-miR-137	000593	LT	
Taqman RNU6b	001093	LT	
Taqman RNU48	001006	LT	

Abbreviations: AB = Applied Biosystems, LT = Life Technologies, IHC = Immunohistochemistry

Supplemental Table 8. Sequences for Oligonucleotide Primers

Genes	Accession #	Sequence (5′-3′)
Musashi-1	NM_002442	F: TTGGCAGACTACGCAGGAAG<br>R: TGGTCCATGAAAGTGACGAAGC
GAPDH	NM_002046	F: ATGTTCGTCATGGGTGTGAA<br>R: GGTGCTAAGCAGTTGGTGGT
Hes-1	NM_005524	F: CTGGAGAGGCGGCTAAGGTGTTT<br>R: GTGCCGCTGTTGCTGGTGTAGA
C-Myc	NM_002467	F: GCCACGTCTCCACACATCAG<br>R: TCTTGGCAGCAGGATAGTCCTT
Pre-miR-137	MI0000454 (miRbase)	F: CGGTGACGGGTATTCTTGGGTGG<br>R: TGCCGCTGGTACTCTCCTCG

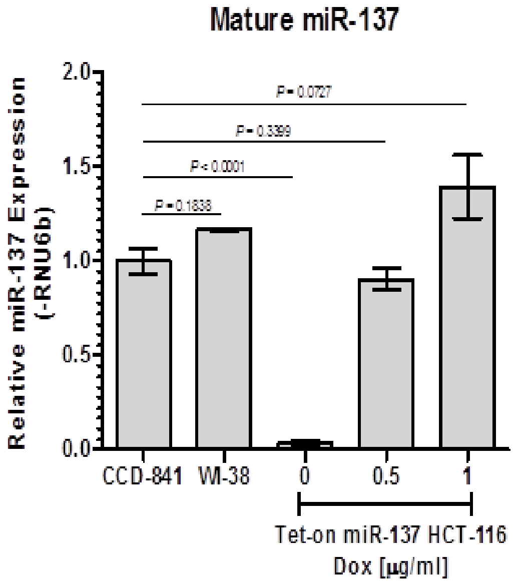

Figure S1. Expression of miR-137 in HCT-116 stable clones (A) Tet-on miR-137 HCT-116 cells were treated with increasing doses of DOX [µg/ml]. Expression of mature miR-137 was analyzed using Taqman qRT-PCR, normalized to RNU6b and set relative to the expression of miR-137 in CCD-841. Expression of miR-137 in WI-38 (lung fibroblast) was also included in the study.
